# Autoimmune polyendocrine syndrome II presenting paradoxically as Takotsubo cardiomyopathy: a case report and reappraisal of pathophysiology

**DOI:** 10.1186/s43044-022-00321-6

**Published:** 2022-12-13

**Authors:** Akash Batta, Amit Kumar Gupta, Gautam Singal, Bishav Mohan, Sushil Kumar, Bhavuk Jaiswal, Juniali Hatwal, Rohit Tandon, Gurbhej Singh, Abhishek Goyal, Bhupinder Singh, Naveen Mittal, Shibba Takkar Chhabra, Naved Aslam, Gurpreet Singh Wander

**Affiliations:** 1 Department of Cardiology, Dayanand Medical College and Hospital, Civil Lines, Ludhiana, 141001 India; 2 Department of Internal Medicine, Advanced Cardiac Centre, Post Graduate Institute of Medical Education & Research, Chandigarh, 160012 India; 3 Department of Cardiology, All India Institute of Medical Sciences, Bathinda, Punjab 151001 India; 4 Department of Endocrinology, Dayanand Medical College and Hospital, Ludhiana, 141001 India

**Keywords:** Addison's disease, Takotsubo cardiomyopathy, Hyperkalemia, Catecholamines, Autoimmune polyendocrine syndrome, Hyponatremia

## Abstract

**Background:**

Takotsubo cardiomyopathy (TCM) is a rare disease entity characterized by acute, non-ischemic, reversible myocardial dysfunction that mimics acute myocardial infarction. Activation and excessive outflow of sympathetic nervous system are believed to be central to the figure in the disease pathogenesis. Adrenocortical hormones potentiate the systemic actions of sympathetic nervous system and accordingly are essential for regulation of myocardial function. We present an unusual case of a middle-aged woman with primary adrenal insufficiency who presented paradoxically with TCM.

**Case presentation:**

A 50-year-old woman with past history of hypothyroidism presented to emergency department with history of acute chest pain and syncope. There was no significant drug history or history of an emotional or physical stimulus prior to admission. Prominent pigmentation over the tongue and skin creases of hands were noted. On presentation, she was in shock and had ventricular tachycardia which required electrical cardioversion. The subsequent electrocardiogram demonstrated diffuse T-wave inversions with prolonged QT_C_. There was apical hypokinesia on echocardiogram, and cardiac biomarkers were elevated. There was persistent inotropic requirement. She had marked postural symptoms, and a postural blood pressure drop of 50 mm Hg was present. Initial laboratory parameters were significant for hyperkalemia (7.8 mEq/L) and hyponatremia (128 mEq/L). These findings prompted evaluation for adrenal insufficiency which was confirmed with appropriate tests. Autoimmune polyendocrine syndrome II was thus diagnosed based on the above findings. Coronary angiography revealed normal coronaries. The diagnoses of TCM was established in accordance with the International Takotsubo Diagnostic Criteria. She was started on stress dose steroid replacement therapy and improved dramatically. At one month of follow-up, the patient is asymptomatic, and there was normalization of her left ventricular function.

**Conclusions:**

Intricate relationship and interplay exist between the steroid hormones and catecholamines in the pathogenesis of TCM. Steroid hormones not only potentiate the actions of catecholamines, but they also regulate and channelize catecholaminergic actions preventing their deleterious effects on the cardiac tissue. Hence, both steroid deficiency and exogenous steroid replacement may precipitate TCM. Evidence from more such cases and larger perspective studies in future will further improve our understanding of this complex disease process and its myriad associations.

**Supplementary Information:**

The online version contains supplementary material available at 10.1186/s43044-022-00321-6.

## Background

Stress-induced cardiomyopathy, also known as Takotsubo cardiomyopathy (TCM), is a rare disease entity characterized by acute, non-ischemic, and reversible myocardial dysfunction that mimics acute myocardial infarction [1–3]. Since the first description in 1990, there has been tremendous increment in the literature surrounding TCM. Despite the obvious progress in our understanding of the disease in the last 3 decades, there are gaps which become apparent when newer associations of this disease are reported. Our case is one such example, wherein a patient of autoimmune polyendocrine syndrome (APS) II presented with TCM.

The most accepted theory on the pathophysiology of TCM is that of catecholaminergic surge secondary to extreme emotional or physiological stress which leads on to myocardial stunning [1, 2, 4]. The other theories include diffuse coronary spasm, transient occlusion of the left anterior descending artery and microvascular dysfunction. Overall, the activation and excessive outflow of sympathetic nervous system are believed to be central to the deleterious effects of the disease [1, 5]. Adrenal cortical hormones have a permissive effect in potentiating the systemic actions of sympathetic nervous system. Corticosteroids besides potentiating the adrenergic effects of catecholamines stimulate the synthesis of norepinephrine and epinephrine [6–8]. Accordingly, the harmful effects of excess catecholamines are likely to be subdued in the absence of adrenocortical steroid hormones.

We present an unusual case of a middle-aged woman with primary adrenal insufficiency who presented with TCM and had a complete recovery after institution of hydrocortisone therapy.

## Case presentation

**Fig. 1:**
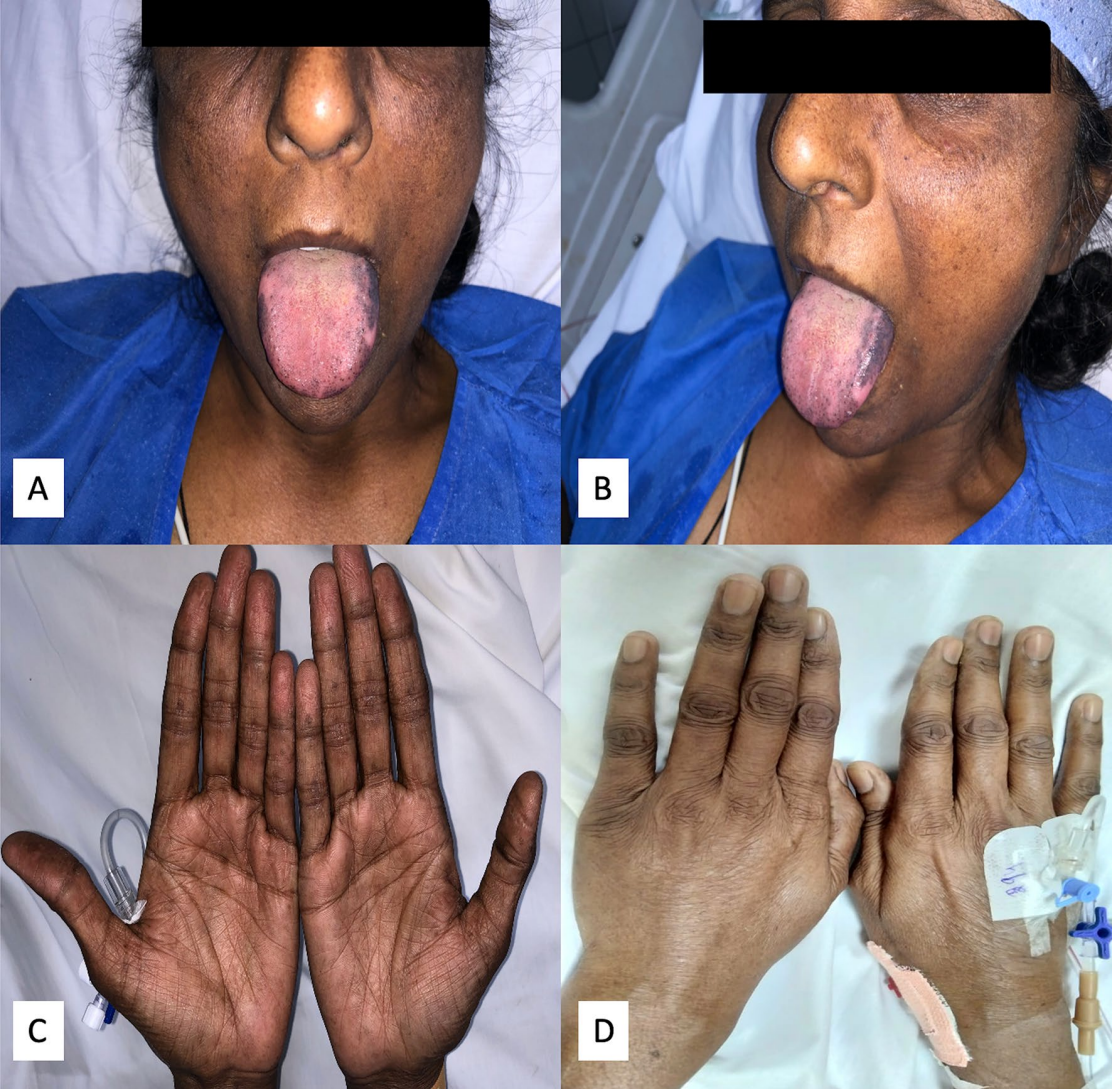
Clinical pictures of the patient showed the patchy hyperpigmentation over the face and the tongue corresponding to the areas of increased friction (**A**, **B**). Skin creases and knuckles of both hands also showed hyperpigmentation (**C**, **D**)

**Fig. 2:**
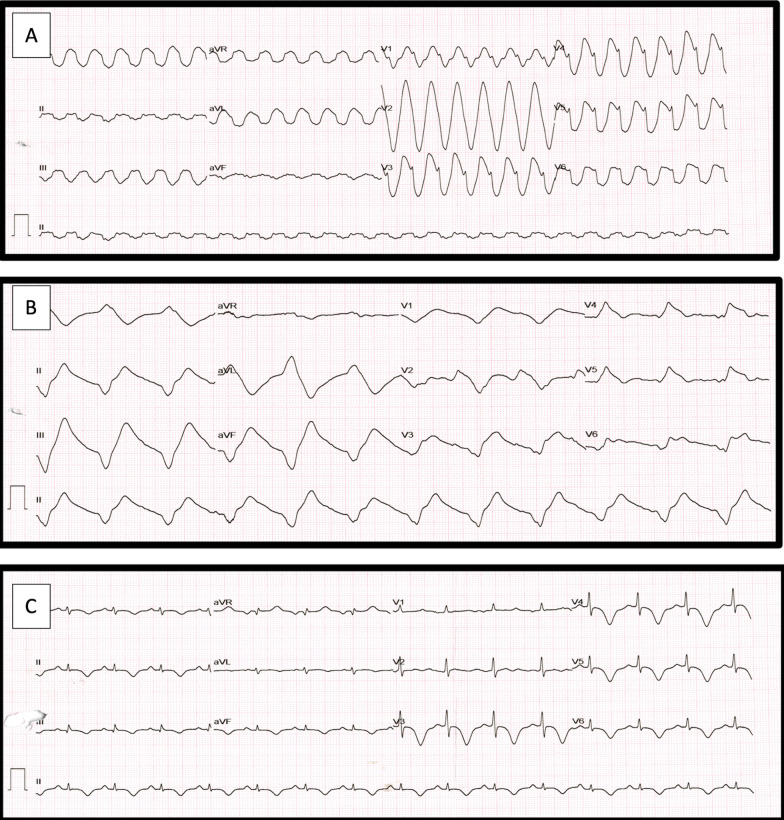
Electrocardiogram of the patient showed monomorphic ventricular tachycardia upon presentation (**A**). Post-electrical cardioversion, there was sine-wave pattern on the electrocardiogram consistent with severe hyperkalemia (**B**). Serum potassium at the time was 7.8 mEq/L. The electrocardiogram after potassium correction revealed T-wave inversions in the anterior and inferior leads with a prolonged QT_C_ of 530 ms (**C**)

A 50-year-old woman with past history of hypothyroidism (on 25 μg levothyroxine) presented to emergency department with history of acute chest pain for 1 day prior to admission and an episode of syncope 15 min prior to admission. There was no significant drug history or history of an emotional or physical stimulus prior to admission. On examination the patient was in shock and had a feeble pulse with a rate of 150/min and a systolic blood pressure of 60 mm of Hg. General physical examination was notable for prominent pigmentation over the tongue and skin creases on hands (Fig. 1). Cardiovascular examination was unremarkable. The initial electrocardiogram revealed monomorphic ventricular tachycardia with a rate of 160/min (Fig. 2A). The same was cardioverted via 200 J biphasic DC shock. Electrocardiogram after cardioversion revealed a sine-wave pattern consistent with hyperkalemia (Fig. 2B). Calcium gluconate and other anti-hyperkalemic measures were instituted, and gradually, there was resolution of the sine-wave pattern. The subsequent electrocardiogram obtained had sinus rhythm with T-wave inversions in anterior and inferior leads with a prolonged QT_C_ of 530 ms (Fig. 2C). Her blood pressure responded to intravenous saline and minimal dose of noradrenaline and subsequently was 120/60 mm of Hg. The urine output was adequate at 100 mL/hour. Bedside echocardiography demonstrated regional wall motion abnormalities with a hypokinetic and ballooned apex corresponding to left anterior descending artery. The left ventricular ejection fraction was 35% with a normal systolic pulmonary artery pressure of 35 mm of Hg (Additional file 1: Video 1). There was no left ventricular outflow gradient. The working diagnosis at this stage was acute anterior wall myocardial infarction, and accordingly dual antiplatelets, statins and low molecular weight heparin were instituted.

The initial laboratory investigations were significant for hyperkalemia (7.8 mEq/L) and hyponatremia (128 mEq/L) with a preserved renal function (creatinine-1.0 mg/dL). There was microcytic and hypochromic anemia with a hemoglobin of 11 mg/dL. The cardiac biomarkers were modestly elevated consistent with acute myocardial injury with a cardiac troponin I of 0.73 ng/mL (range 0.0–0.03 ng/mL). Her thyroid function tests were also deranged with an elevated thyroid-stimulating hormone of 67.70 mIU/L [1–3] and low T3 (2 pmol/L) and T4 (10 pmol/L). Despite adequate anti-hyperkalemic measures, the serum potassium remained elevated (5.8–6.5 mEq/L) for the next 48 h; however, the electrocardiogram changes of hyperkalemia had reverted.

The patient was managed conservatively for the next 24 h but still required persistent inotropic support and intravenous fluids for maintenance of blood pressure. There was significant postural dizziness, and a postural fall of 50 mm of Hg was present at 3 min. Her levothyroxine was escalated to 75 μg daily. In view of these postural symptoms, skin changes and significant electrolyte abnormalities in the background of normal renal parameters, suspicion of adrenal insufficiency was kept, and investigations were performed accordingly. The subsequent 8 am cortisol was 0.429 mcg/dL consistent with adrenal insufficiency. To identify the cause of adrenal insufficiency, a cosyntropin test was performed, and the subsequent 8 am cortisol was 0.423 mcg/dL consistent with primary adrenal insufficiency. The diagnosis of APS II was established.

**Fig. 3:**
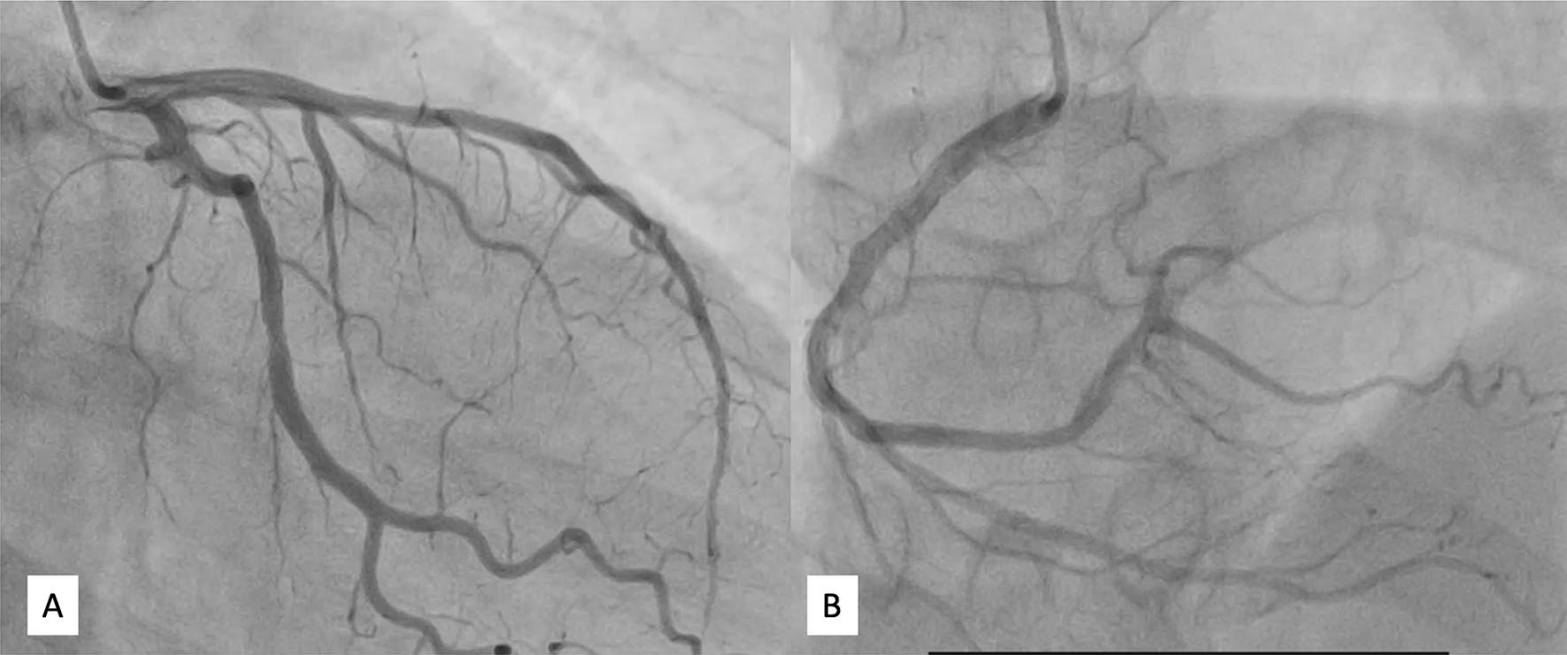
Still frames of coronary angiogram demonstrating normal coronaries (**A**, **B**)

The patient had remarkable improvement in her hemodynamics after initiation of intravenous 50 mg hydrocortisone every 6 h. The postural blood pressure fall resolved after 48 h and hyperkalemia also resolved. To rule out coexistent coronary artery disease, an invasive coronary angiogram was performed which revealed normal coronaries and the presence of apical hypokinesia on left ventricular angiogram (Fig. 3 and Additional file 2: Video 2). The echocardiogram showed improvement in cardiac function with an ejection fraction of 45% and regional wall motion abnormalities. The cardiac troponin I fell to 0.13 ng/mL (range 0.0–0.03 ng/mL). The diagnoses of TCM were established in accordance with the International Takotsubo Diagnostic Criteria [3]. Antiplatelets and statins were subsequently withdrawn, and she was discharged on 50 mg metoprolol, oral prednisolone 10 mg twice daily and 75 μg levothyroxine.

At one month of follow-up, the patient was asymptomatic and there was normalization of her left ventricular ejection fraction (Additional file 3: Video 3). Currently, the patient is doing fine at 6 months of follow-up.

## Discussion

TCM is characterized by a self-limiting myocardial dysfunction with complete recovery in most cases within weeks of diagnosis. Though the vast majority has an identifiable emotional or physical trigger prior to the illness, up to one-third have no specific trigger [1, 5]. The disease in general has good long-term prognosis with an in-hospital mortality of around 4%. The high-risk features include left ventricular ejection fraction < 45%, a left ventricular outflow gradient > 40 mm of Hg and the presence of cardiac arrhythmia. Most patients do not have associated coronary artery disease; however, the presence of a significant coronary artery disease does not exclude the diagnosis of TCM. The recurrence rate is maximum within the first year and is around 5% [1–3, 5, 9].

The International Takotsubo Diagnostic Criteria have been developed to aid in the diagnosis of TCM. The most important components of the criteria are transient left ventricular dysfunction, the presence of an emotional or physical trigger prior to the episode, new electrocardiographic abnormalities, moderately elevated cardiac biomarkers and no evidence of infectious myocarditis. Accordingly, an InterTAK clinical score was also developed to differentiate TCM from acute coronary syndromes [3, 9].

APS II is a rare endocrine disorder characterized by the coexistence of autoimmune thyroid disease with Addison’s disease and/or diabetes mellitus. Majority of the case of TCM complicating Addison’s disease have been reported in the pediatric age group. The exact pathogenesis of TCM remains elusive, but activation of sympathetic nervous system is believed to be the central theme. Excess catecholamines have deleterious effects on the heart resulting in acute myocardial dysfunction [10]. The proposed theories for catecholamine-mediated myotoxicity include the overstimulation of beta-adrenergic receptors which results in a positive inotropic and chronotropic effect leading to imbalance in oxygen supply demand and resultant ‘functional’ hypoxia [7, 11]. Further, excessive stimulation of the alpha receptors on the coronary arteries causes vasospasm and vasoconstriction causing reduced blood supply and myocardial ischemia and dysfunction [6–8]. Other postulations include a downregulation of the beta-adrenergic receptors because of persistent stimulation eventually culminating in reduced myocyte function, oxidative stress, altered calcium homeostasis in the myocardial cells and mitochondrial dysfunction. The same is exemplified by the close association of TCM with pheochromocytoma— another condition in which catecholamine excess results in acute myocardial dysfunction [12].

Abundant literature points to the permissive effects of cortisol in synthesis of catecholamines as well as facilitating the action of catecholamines on heart and vascular smooth muscles. It enhances the inotropic actions of catecholamines and facilitates increased vasoconstriction by the sympathetic nervous system [7, 8]. Unsurprisingly, there has been association of TCM during induction of steroid therapy for unrelated illnesses reflecting the enhanced actions of catecholamines at alpha and beta receptors [13]. This association can be explained by the improved sensitivity of the cardiac myocardium to the adrenergic system and an exaggerated catecholamine-induced myocardial dysfunction. Another similar association is the occurrence of TCM during administration of higher doses of levothyroxine among hypothyroid patients. The same is also attributable to the permissive effect of thyroid hormones in potentiating the action of catecholamines [4, 14].

The paradox in the index case is the development of TCM in a patient with adrenal insufficiency, wherein in the absence of steroid hormones, the toxic effects of sympathetic catecholamines were realized. Indeed, there have been only very few case reports documenting this association to date [15–17]. Pathophysiological mechanism of this association is largely unknown, but certain theories have been hypothesized. In adrenalectomized animal models, there was a toxic effect of catecholamines on the cardiac tissue when they were exposed to stress. The other theories include uncoupling of excitation–contraction in the myocardial cells in the absence of steroid hormones [4]. Hence, the steroid hormones have variable actions on the sympathetic nervous system with both permissive and regulatory effects which is essential for positive inotropic and chronotropic effects on the myocardium in healthy individuals. Deviation from this finely balanced milieu results in myocardial dysfunction as seen in TCM.

TCM is usually a self-limiting disease, and treatment is aimed at achieving early resolution of myocardial dysfunction and preventing future recurrence. Angiotensin-converting enzyme inhibitors have shown promising results in this aspect and are indicated for most patients with TCM [1, 2]. Betablockers are not needed in the majority of cases though some evidence points toward its role in nullifying the sympathetic surge associated with TCM and may help in clinical improvement. In the index case, the induction of stress dose glucocorticoid therapy had a favorable effect on the myocardium. The same has been reported earlier although sparingly [15–18]. This may reflect the role of glucocorticoids in proper functioning of the membrane calcium transport chain in the sarcoplasmic reticulum which is essential for adequate myocardial contractility [4]. However, one must remember that glucocorticoid therapy initiation is a double-edged sword and it may have an unfavorable effect on myocardial contractility in some patients as hypothesized above. To the best of our knowledge, this is only the 5th case report in the literature in which TCM was associated with APS II [19–22].

## Conclusions

In conclusion, the evidence from our case and prior cases reported in the literature linking TCM to Addison’s disease points to the intricate relationship and interplay between the steroid hormones and catecholamines in the pathogenesis of TCM. Steroid hormones not only potentiate the actions of catecholamines, but they also regulate and channelize their actions preventing the deleterious effects on the cardiac tissue. Hence, both steroid deficiency and exogenous steroid replacement may precipitate TCM. Further cases and evidence from larger perspective studies in future will further improve our understating of this complex disease processes.

## Supplementary Information

**Additional file 1: Video 1**: Apical 4 chamber view of the patient showed severe apical hypokinesia and a left ventricular ejection fraction of 35% upon admission.

**Additional file 2: Video 2**: Left ventricular contrast angiogram also showed reduced left ventricular apical contractility.

**Additional file 3: Video 3**: Apical 4 chamber view of the patient at one-month follow-up showed normalization of left ventricular ejection fraction and resolution of apical hypokinesia.

## Data Availability

All data and materials will be uploaded as per the needs of the editor/reviewer or the readers as per their request.

## Abbreviations

TCM: Takotsubo cardiomyopathy
APS: Autoimmune polyendocrine syndrome
